# A Fast and Robust Non-Sparse Signal Recovery Algorithm for Wearable ECG Telemonitoring Using ADMM-Based Block Sparse Bayesian Learning

**DOI:** 10.3390/s18072021

**Published:** 2018-06-23

**Authors:** Yunfei Cheng, Yalan Ye, Mengshu Hou, Wenwen He, Yunxia Li, Xuesong Deng

**Affiliations:** 1 School of Computer Science and Engineering, University of Electronic Science and Technology of China, Chengdu 611731, China; yunfeicheng@hotmail.com (Y.C.); mshou@uestc.edu.cn (M.H.); hwwuestc@gmail.com (W.H.); yingkesongsxd@gmail.com (X.D.); 2 School of Automation Engineering, University of Electronic Science and Technology of China, Chengdu 611731, China; yunxiali@uestc.edu.cn

**Keywords:** wireless body area networks (WBAN), electrocardiogram (ECG), compressed sensing (CS), block sparse Bayesian learning (BSBL), alternating direction method of multipliers (ADMM)

## Abstract

Wearable telemonitoring of electrocardiogram (ECG) based on wireless body Area networks (WBAN) is a promising approach in next-generation patient-centric telecardiology solutions. In order to guarantee long-term effective operation of monitoring systems, the power consumption of the sensors must be strictly limited. Compressed sensing (CS) is an effective method to alleviate this problem. However, ECG signals in WBAN are usually non-sparse, and most traditional compressed sensing recovery algorithms have difficulty recovering non-sparse signals. In this paper, we proposed a fast and robust non-sparse signal recovery algorithm for wearable ECG telemonitoring. In the proposed algorithm, the alternating direction method of multipliers (ADMM) is used to accelerate the speed of block sparse Bayesian learning (BSBL) framework. We used the famous MIT-BIH Arrhythmia Database, MIT-BIH Long-Term ECG Database and ECG datasets collected in our practical wearable ECG telemonitoring system to verify the performance of the proposed algorithm. The experimental results show that the proposed algorithm can directly recover ECG signals with a satisfactory accuracy in a time domain without a dictionary matrix. Due to acceleration by ADMM, the proposed algorithm has a fast speed, and also it is robust for different ECG datasets. These results suggest that the proposed algorithm is very promising for wearable ECG telemonitoring.

## 1. Introduction

Wearable telemonitoring of electrocardiogram (ECG) via wireless body area networks (WBAN) is a very important topic in telemedicine, and many works focused on the devices, sensor networks and other subjects in ECG telemonitoring [1,2,3], but the most significant challenge of practical application of wearable remote ECG monitoring system is power consumption [4]. Some researchers have demonstrated that the main consumption of energy in WBAN is data acquisition and wireless data transmission [5,6]. In common WBAN-based ECG monitors, sensor nodes fall short of energy efficiency due to large data acquired from continuous monitoring and the energy wireless links [7,8], thus it is desirable to reduce the amount of data that need to be acquired and transmitted.

**Figure 1:**
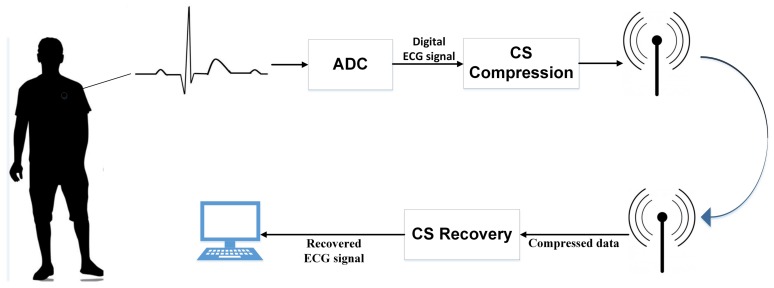
Typical compressed sensing (CS)-based ECG wireless telemonitoring system.

Compressed sensing (CS) [9,10,11] is an emerging signal sampling technique that requires a much lower sampling rate to recover the original signal compared with the traditional Nyquist sampling method. Figure 1 shows a typical CS-based wireless ECG telemonitoring system, where the ECG signals are firstly sampled via an analog-digital converter; then, these samples are compressed by a measurement matrix and transmitted to the remote terminal, and the ECG signals are recovered from the compressed data in the remote terminal. Many studies have focused on the application of compressed sensing in WBAN-based ECG monitoring [12,13,14,15], but it is still in its infancy [16].

Generally, the recorded ECG signals in wireless telemonitoring are non-sparse in the time domain and it is hard to find other sparse domains [17] for signal recovery, while compressed sensing algorithms usually assume that the signals are sparse in a time domain or some other transformed domains and the signals are recovered in the sparse domain. Most common compressed sensing recovery algorithms [18,19,20,21,22,23] usually can accurately recover sparse signals, but can not do much about non-sparse signals [24]. Therefore, accurate recovery of non-sparse signals is very important for wearable ECG telemonitoring.

In order to solve the problem of compressed sensing recovery on non-sparse signals, Zhang et al. [25,26] proposed a block sparse Bayesian learning (BSBL) framework-based BSBL-BO (bound optimization) algorithm to accurately recover non-sparse physiological signals. Exploiting the intra-block correlation of the signals, the BSBL framework has shown its outstanding performance on physiological signals. However, for the practical wearable wireless ECG monitoring system, BSBL-BO is too slow to recover the ECG signals in time. Zhang et al. [25] have proposed another BSBL-L1 algorithm that is very fast, but its recovery accuracy is much worse than BSBL-BO. Similarly, Liu et al. [27] proposed a fast algorithm based on the BSBL framework while the recovery accuracy is also unsatisfactory. Block sparse Bayesian learning algorithms usually can recover non-sparse directly by exploiting the intra-block correlation of signals such as BSBL-BO [25], but some other BSBL-based algorithms can not recover non-sparse signals accurately such as BSBL-L1 [25] and BSBL-FM [27]. In fact, most existing compressed sensing algorithms fail to meet the requirements of real-time ECG telemonitoring for speed and accuracy. Therefore, a fast algorithm that can accurately recover non-sparse signals is very necessary.

In this work, in order to recover non-sparse ECG signal in wireless ECG monitoring fast and accurately, a fast and robust non-sparse signal recovery algorithm termed BSBL-ADMM based on BSBL framework was proposed. The minimization of BSBL cost function was transformed to a group lasso optimization problem and alternating direction multiplier method (ADMM) [28] is used to solve the optimization problem because of that ADMM can make the proposed algorithm jumpily converged. The proposed BSBL-ADMM algorithm can directly recover the non-sparse ECG signals in the time domain quickly and satisfactory accuracy. We used the famous MIT-BIH Arrhythmia Database [29] and MIT-BIH Long-Term ECG Database [30] to demonstrate the outstanding performance of the proposed BSBl-ADMM algorithm. In addition, we also built a practical wearable ECG telemonitoring system based on digital compressed sensing and the proposed BSBL-ADMM algorithm is simulated on the ECG datasets collected in our practical wearable ECG telemonitoring system. The experimental results show that the proposed BSBL-ADMM algorithm not only has the ability to recover non-sparse ECG signals with fast speed and satisfactory recovery accuracy, but is also robust against different ECG datasets. Therefore, the peoposed BSBL-ADMM algorithm is promising for telemonitoring of ECG signals. The main contributions of this paper are as follows:

- While the sparse domain of ECG signals and the corresponding dictionary matrix are hard to find, the proposed BSBL-ADMM algorithm has ability to directly recover the non-sparse ECG signal in a time domain without a dictionary matrix, instead of some CS algorithms that need to transform the signal to a sparse domain by a dictionary matrix. Therefore, the proposed BSBL-ADMM algorithm is suitable for non-sparse ECG signal recovery in wearable ECG telemonitoring.
- The alternating direction multiplier method (ADMM) is introduced in BSBL framework to solve the optimization problem of cost function. ADMM can reach an approximate iteration result quickly in just a few iterations that make the proposed algorithm jumpily converged. Thus, the proposed BSBL-ADMM algorithm can meet the real-time requirement of the wearable ECG telemonitoring.
- To ensure the generality of the proposed algorithm, a practical wearable ECG telemonitoring system based on digital compressed sensing is built to collect practical wearable ECG datasets. The proposed algorithm was simulated on three different datasets include our practical wearable ECG datasets and two MIT-BIH Databases. The experimental results demonstrate the outstanding performance and robustness of the proposed BSBL-ADMM algorithm. These advantages make the application of the proposed BSBL-ADMM algorithm in wearable ECG telemonitoring to be very promising.

The remainder of this paper is organized as follows: Section 2 states the block sparse Bayesian learning (BSBL) framework; Section 3 presents the proposed BSBL-ADMM algorithm; Section 4 describes datasets, experiment settings, and experimental results; conclusions are given in the last section.

## 2. BSBL Framework for ECG Recovery

To facilitate the study of algorithms, we firstly assume that signals have passed through the analog-digital converter (ADC), as in the “digital CS” paradigm [13].

### 2.1. Compressed Sensing Based on WBAN

Compressed sensing is a newly introduced data sampling and compression technique in wearable ECG telemonitoring based on wireless body area networks.

Most compressed sensing algorithms recover the signals in a sparse transformed domain. However, in WBAN, the signals are usually non-sparse and it is also hard to find a sparse transformed domain. The Block Sparse Bayesian Learning (BSBL) algorithms [25] can usually recover the non-sparse ECG signals in the time domain directly.

The original ECG signal is denoted by α; then, the basic model of CS can be expressed as

(1) s=Φα,

where *s* is the compressed data and Φ is the measurement matrix whose row number is smaller than column number; both *s* and Φ are known to CS algorithm for recovery. CS algorithms use the compressed data *s* and the measurement matrix Φ to recover the original ECG signal α.

### 2.2. Block Sparse Bayesian Learning

In applications, α generally has some structures, and a widely studied structure is the block structure. With this structure, α can be expressed as:

(2) $$\alpha ={\left[\underset{\alpha_{1}^{T}}{\underset{︸}{\alpha_{1},\cdots ,\alpha_{d_{1}}}},\cdots ,\underset{\alpha_{g}^{T}}{\underset{︸}{\alpha_{d_{g-1}+1},\cdots ,\alpha_{d_{g}}}}\right]}^{T}.$$

It means that α has *g* blocks, and, among these blocks, only a few blocks are nonzero. Of course, if the signal is non-sparse, then the intra-block correlation will determine whether the signal can be recovered successfully. In BSBL [25], each block $\alpha_{i}$ is modeled as a parameterized multivariate Gaussian distribution:

(3) $$p\left(\alpha_{i};\gamma_{i},B_{i}\right)=N\left(\alpha_{i};0,\gamma_{i}B_{i}\right),$$

where $\gamma_{i}$ is an unknown nonnegative parameter controlling the block-sparsity of α, and $B_{i}$ is an unknown positive definite matrix modeling the intra-block correlation structure of the block $\alpha_{i}$. The blocks are assumed to be mutually independent; then, the prior of α is $p\left(\alpha ;\left\{\gamma_{i},B_{i}\right\}\right)=N\left(0,\Sigma_{0}\right)$, where $\Sigma_{0}=diag\left\{\gamma_{1}B_{1},\cdots ,\gamma_{g}B_{g}\right\}$. Assume the measurement noise to be independent and satisfy a Gaussian distribution with zero mean and unknown variance λ, thus the Gaussian likelihood is

(4) p(s|α;λ)=N(s;Φα,λI).

In addition, the posterior of x is expressed as

(5) $$p\left(\alpha |s;{\left\{\gamma_{i},B_{i}\right\}}_{i=1}^{g},\lambda \right)=N\left(\mu ,\Sigma \right),$$

where

(6) $$\mu =\Sigma_{0}\Phi^{T}{\left(\lambda I+\Phi \Sigma_{0}\Phi^{T}\right)}^{-1}s,$$

(7) $$\Sigma ={\left(\Sigma_{0}^{-1}+\frac{1}{\lambda }\Phi^{T}\Phi \right)}^{-1}.$$

The parameters can be estimated by a Type II maximum likelihood procedure, which is equivalent to minimize the following cost function:

(8) $$L\left({\left\{\gamma_{i},B_{i}\right\}}_{i},\lambda \right)=log\left|\lambda I+\Phi \Sigma_{0}\Phi^{T}\right|+s^{T}{\left(\lambda I+\Phi \Sigma_{0}\Phi^{T}\right)}^{-1}s.$$

This cost function can be minimized by many optimization methods such as expectation maximization (EM), bound optimization (BO) [25] and fast marginal likelihood maximization (FM) [27]. The EM and BO algorithms can achieve a high accuracy on non-sparse signal recovery while the speed is slow. The FM algorithm is much faster than EM and BO, but the recovery accuracy of FM is not satisfactory.

## 3. Proposed ADMM-Based BSBL

In order to make a fast CS recovery algorithm based on the BSBL framework [25], the cost function will be transformed from a function of γ to a function of *x*. We introduce the alternating direction multiplier method (ADMM) to solve the minimization of the transformed cost function. The iteration of ADMM is the inner loop of the proposed algorithm, and it need not converge completely and an approximate iteration result can make the proposed algorithm jumpily converged. ADMM can get a approximate iteration result quickly in just a few iterations and the completely converged result has little influence to the final recovery accuracy, thus the main loop of the proposed algorithm can converge rapidly.

### 3.1. Transformation of the Cost Function

The BSBL cost function (8) can be transformed from γ-space to *x*-space using some identity and inequality. The parameters λ and *B* can be treated as fixed values first for convenience.

As presented in Zhang et al. [25], the minimization of cost function can be transformed to the following optimization

(9) $$\alpha^{k+1}=\arg\min_{\alpha }{∥s-\Phi \alpha ∥}_{2}^{2}+\lambda \sum_{i}\sigma_{i}^{\left(k\right)}\sqrt{\alpha_{i}^{T}B_{i}^{-1}\alpha_{i}}\;,$$

where $\sigma_{i}=2\;\;{\left(Tr\left[B_{i}{\Phi^{i}}^{T}{\left(\lambda I+\Phi \Sigma_{0}\Phi^{T}\right)}^{-1}\Phi^{i}\right]\right)}^{\frac{1}{2}}$, and $\gamma_{i}=\frac{2\sqrt{\alpha_{i}^{T}B_{i}^{-1}\alpha_{i}}}{\sigma_{i}}$.

In addition, the learning rule of λ and $B_{i}$ can be derived using the EM method

(10) $$\lambda =\frac{||s-{\Phi \alpha ||}_{2}^{2}+Tr\left(\Sigma \Phi^{T}\Phi \right)}{M},$$

(11) $$B_{i}=\frac{\Sigma^{i}+\mu^{i}{\left(\mu^{i}\right)}^{T}}{\gamma_{i}},$$

where *M* is the length of α, $\mu^{i}$ is the corresponding *i*-th block in μ, and $\Sigma^{i}$ is the corresponding *i*-th principal diagonal block in Σ.

Then, in order to avoid overfitting, a strategy including parameter average and the first-order auto-regressive (AR) process is used to constrain each $B_{i}$ to be the same matrix *B*. The average of all $B_{i}$ is calculated as

(12) $$\bar{B}=\frac{1}{g}\sum_{i=1}^{g}B_{i}\;,$$

then the matrix *B* can be reconstructed by a Toeplitz matrix as

(13) $$B=Toeplitz(\left[1,r,\cdots ,r^{d-1}\right]),$$

where *r* is the AR coefficient empirically calculated by $r=r_{1}/r_{0}$, in which the $r_{0}$ and $r_{1}$ are the average of the elements along the main diagonal and main sub-diagonal of the matrix $\bar{B}$.

### 3.2. Minimizing the Cost Function Using ADMM

As the minimization of BSBL cost function (8) was transformed to the optimization problem (9), it is easy to find that the problem (9) can be transformed into a L1/L2 regularization, which is called group lasso [31]. Let $u_{i}=\sigma_{i}B_{i}^{-1/2}\alpha_{i}$, $u={\left[u_{1}^{T},u_{2}^{T},\cdots ,u_{g}^{T}\right]}^{T}$, and $H=\Phi \cdot diag\{B_{1}^{1/2}/\sigma_{1},\cdots ,B_{g}^{1/2}/\sigma_{g}\}$, then the problem (9) can be transformed to the following form:

(14) $$\begin{array}{c} u^{\left(k+1\right)}=\arg\min_{u}{∥s-Hu∥}_{2}^{2}+\lambda \sum_{i}{∥u_{i}∥}_{2},\\ \;\;\;\;\;\;\;\;\propto \arg\min_{u}\frac{1}{2}{∥s-Hu∥}_{2}^{2}+\lambda_{0}\sum_{i}{∥u_{i}∥}_{2},\; \end{array}$$

where $\lambda_{0}=\frac{1}{2}\lambda$.

Furthermore, it can be seen that problem (14) has a similar form as ADMM framework [28], then let $f\left(u\right)=\frac{1}{2}||s-{Hu||}_{2}^{2}$, $g\left(z\right)=\lambda_{0}\sum_{i}\left|\right|z_{i}|{|}_{2}$, the problem (9) can be expressed as an ADMM form:

(15) $$\left\{\begin{array}{c} minimize\;\;f(u)+g(z),\\ subject\;to\;\;u-z=0.\;\;\; \end{array}\right.$$

### 3.3. Solving the ADMM

ADMM is an algorithm that solves convex optimization problems by breaking them into smaller pieces, each of which are then easier to handle. As in the method of multipliers, the form of the augmented Lagrangian is as the following:

(16) $$L_{\rho }\left(u,z,b\right)=f\left(u\right)+g\left(z\right)+b^{T}\left(u-z\right)+\left(\rho /2\right)||u-{z||}_{2}^{2}\;.$$

Then, the ADMM consists of the iterations

(17) $$u^{j+1}=\arg\min_{u}L_{\rho }\left(u,z^{j},b^{j}\right),\;\;\;\;$$

(18) $$z^{j+1}=\arg\min_{z}L_{\rho }\left(u^{j+1},z,b^{j}\right),$$

(19) $$b^{j+1}=b^{j}+\rho \left(u^{j+1}-z^{j+1}\right),\;\;\;\;$$

where ρ>0. In ADMM, the *u* and *z* are updated in an alternating fashion, which accounts for the term alternating direction.

The ADMM also can be written in a scaled form, which is more convenient. Defining the residual r=u−z, then

(20) $$\begin{array}{cc} b^{T}\left(u-z\right)+\left(\rho /2\right)||u-{z||}_{2}^{2} & =b^{T}r+{\left(\rho /2\right)||r||}_{2}^{2}\\ & =\left(\rho /2\right)||r+{\left(1/\rho \right)b||}_{2}^{2}-{\left(1/2\rho \right)||b||}_{2}^{2}\\ & =\left(\rho /2\right)||r+{y||}_{2}^{2}-{\left(\rho /2\right)||y||}_{2}^{2}, \end{array}$$

where y=(1/ρ)b is the scaled dual variable. The ADMM can be expressed as:

(21) $$u^{j+1}=\arg\min_{u}\left(\frac{1}{2}||s-{Hu||}_{2}^{2}+\left(\rho /2\right)||u-z^{j}+y^{j}{\left|\right|}_{2}^{2}\right),\;\;\;\;\;\;$$

(22) $${z_{i}}^{j+1}=\arg\min_{z_{i}}\left(\lambda_{0}\sum_{i}\left|\right|z_{i}|{|}_{2}+\left(\rho /2\right)||u^{j+1}-z_{i}+y^{j+1}{\left|\right|}_{2}^{2}\right),$$

(23) $$y^{j+1}=y^{j}+u^{j+1}-z^{j+1}.\;\;\;\;\;\;\;\;\;\;\;\;\;\;\;\;\;\;\;\;\;\;\;\;\;\;\;\;\;\;\;\;\;\;\;\;\;\;\;\;\;\;\;\;\;\;\;\;\;\;\;\;\;\;\;$$

The step over *u* (21) is an unconstrained strongly convex quadratic minimization whose unique solution is given by a linear system as follows:

(24) $$u^{j+1}={\left(H^{T}H+\rho I\right)}^{-1}\left(H^{T}s+\rho \left(z^{j}-y^{j}\right)\right).$$

The step over $z_{i}$ (22) has a simple closed-form solution as

(25) $${z_{i}}^{j+1}=S_{\lambda_{0}/\rho }\left({u_{i}}^{j+1}+y^{j}\right),$$

where the soft thresholding operator *S* is defined as

(26) $$S_{k}\left(a\right)\left\{\begin{array}{c} a-k,\;\;\;\;\;a>k,\;\;\;\;\\ 0,\;\;\;\;\;\;|a|\;<k,\\ a+k,\;\;\;\;a<-k.\;\;\; \end{array}\right.$$

The *u*, *z* and *y* are iteratively updated by (23)–(25) and until converging. Using ADMM to solve the problem (14), all the subproblems are easy to solve, and the algorithm does not need strict conditions to ensure the global convergence. In addition, in our problem, it need not converge completely because the result with complete convergence has little effect on the proposed algorithm. Once the $u_{i}$ is solved, the $\alpha_{i}$ can be obtained by

(27) $$\alpha_{i}=B_{i}^{1/2}\sigma_{i}^{-1}u_{i}\;.$$

Note that, through the approximate iteration result that was reached in just a few iterations, the ADMM makes the proposed algorithm jumpily converged, thus the proposed algorithm can get a fast recovery speed. The following Algorithm 1 gives the main procedure of the proposed algorithm, which we call the BSBL-ADMM algorithm.
**Algorithm 1 Proposed BSBL-ADMM Algorithm**1:Input: Φ,s2:Initialize: $\alpha =\left[1,1,\ldots ,1\right],B_{i}=I,\sigma_{i}=1$3:While not converge4:**while** not converge **do**5: Calculate $\gamma_{i}=\frac{2\sqrt{\alpha_{i}^{T}B_{i}^{-1}\alpha_{i}}}{\sigma_{i}}$6: Calculate $\lambda =\frac{||s-{\Phi \alpha ||}_{2}^{2}+Tr\left(\Sigma \Phi^{T}\Phi \right)}{M}$7: Calculate $B_{i}=\frac{\Sigma^{i}+\mu^{i}{\left(\mu^{i}\right)}^{T}}{\gamma_{i}}$8: Calculate $B=Toeplitz(\left[1,r,\cdots ,r^{d-1}\right])$9: Calculate $\sigma_{i}=2\;\;{\left(Tr\left[B_{i}{\Phi^{i}}^{T}{\left(\lambda I+\Phi \Sigma_{0}\Phi^{T}\right)}^{-1}\Phi^{i}\right]\right)}^{\frac{1}{2}}$10: Calculate $H=\Phi \cdot diag\{B_{1}^{1/2}/\sigma_{1},\cdots ,B_{g}^{1/2}/\sigma_{g}\}$11: **while** not converge **do**12:  Calculate $u^{j+1}={\left(H^{T}H+\rho I\right)}^{-1}\left(H^{T}s+\rho \left(z^{j}-y^{j}\right)\right)$13:  Calculate ${z_{i}}^{j+1}=S_{\lambda_{0}/\rho }\left({u_{i}}^{j+1}+y^{j}\right)$14:  Calculate $y^{j+1}=y^{j}+u^{j+1}-z^{j+1}$15: **end while**16:**end while**17:Calculate $\alpha_{i}=B_{i}^{1/2}\sigma_{i}^{-1}u_{i}$18:Output: α

## 4. Experiments and Results

### 4.1. Datasets

In order to validate the performance of the proposed BSBL-ADMM algorithm without loss of generality, we used three different datasets including the famous MIT-BIH Arrhythmia Database [29], MIT-BIH Long-Term ECG Database [30] and the practical wearable ECG datasets as the original signals to generate the compressed data.

**Figure 2:**
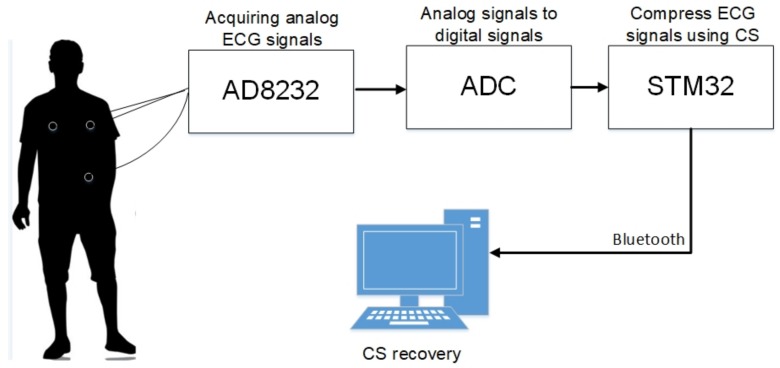
Block diagram of a wearable ECG telemonitoring system.

**Figure 3:**
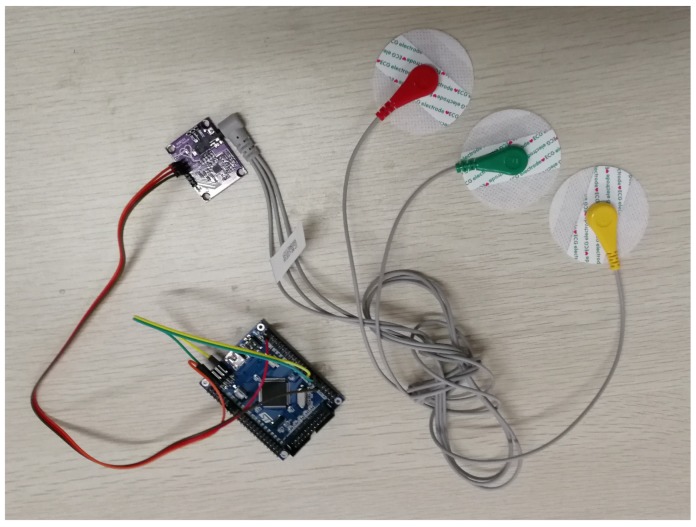
Devices of a wearable ECG collecting system.

(1) MIT-BIH Arrhythmia Database: This is the most representative database for arrhythmia, and as such it has been used for most of the published research. It was also the first database available for analysis of the ECG signals and has been constantly refined over the years [29,32]. It includes 48 two-channel recordings at 360 samples per second with about 30 min.
(2) MIT-BIH Long-Term ECG Database: This database contains seven long-term ECG recordings, and each recording lasts about 14 h to 22 h at the sampling rate of 128 Hz [30,33], and the long-term ECG recordings can verify the performance of the algorithms effectively.
(3) Practical wearable ECG datasets: We built a practical wearable system for ECG collecting based on digital compressed sensing. Figure 2 and Figure 3 show the block diagram and the practical devices of the ECG collecting system, respectively. In this system, three-lead electrodes are used to obtain ECG signals via AD8232 module [34], the leads LA and RA are attached at the left and right chest respectively and the LL lead is attached at the left lower abdomen. After we obtain the analog ECG signals via AD8232, an analog-digital converter is used to sample the analog signals to digital signals at the sampling frequency 250 Hz [35], which is commonly used for ECG monitoring in body area networks. Then, a STM32 microcontroller is used to compress the digital ECG signals by a simple matrix-vector multiplication based on compressed sensing, these compressed data are transmitted to a computer via bluetooth, and the ECG signals are recovered from the compressed data on the computer. Finally, eight one-channel recordings that lasted about 30 min were collected using this system from eight different people.

### 4.2. Experimental Setup

In our experiments, the original signals were divided into several frames with the length N=500. To obtain the compressed measurements of the length *M*, a measurement matrix must be constructed. Here, we use a sparse binary matrix as the measurement matrix. Note that the sparse binary matrix has been widely used in CS-based telemonitoring for its efficiency in storage and matrix-vector multiplication. The compression ratio (CR) was defined as

(28) $$CR=\frac{N-M}{N}.$$

In the experiments, we first constructed the measurement matrices with N=500 and M=100,150,200,250,300, the corresponding CR=80%,70%,60%,50%,40%. Every measurement matrix was generated with *M* rows and *N* columns. Regardless of the size, its each column contained 12 entries of 1 s with random locations and other entries are all 0 s. Then, the prepared frame vector *x* was multiplied by the measurement matrix and the measurement vector was obtained. Then, the signal would be recovered by the recovery algorithm using the measurement matrix and measurement vector.

### 4.3. Performance Metrics

To measure recovery quality, we use the percentage root-mean squared distortion (PRD) to quantify the error percentage between the original signal α and the recovered signal $\hat{\alpha }$:

(29) $$PRD=\frac{\left|\right|\hat{\alpha }-\alpha |{|}_{2}}{\left||\alpha \right|{|}_{2}}\times 100.$$

The lower PRD means the better recovery performance. In addition, Pearson correlation between original signal and recovered signal is another performance index that reflects the reconstruction quality that higher Pearson correlation means better recovery performance.

Furthermore, the CPU time is used as the performance index, which reflects the speed of algorithms. The computer used in the experiments is a laptop equipped with an Intel Core-i7 6500U (Intel Corporation Co., Ltd., Santa Clara, CA, USA) at 2.50 GHz, 8-GB RAM, Windows 10 64-bit, and MATLAB R2016a (MathWorks, Inc., Natick, MA, USA).

### 4.4. The Compared Algorithms

In order to demonstrate the performance improvement of the proposed algorithm for BSBL framework, we compared it with some classical BSBL-based algorithms including BSBL-BO [25], BSBL-L1 [25] and BSBL-FM [27], in addition to another typical CS algorithm Accelerated Iterative Hard Thresholding (AIHT) [19], which is used for comparison in order to show the advantage of BSBL framework only for sparse signal recovery. All of these algorithms would be used to recover the ECG signals directly without a dictionary matrix.

### 4.5. Results on MIT-BIH Arrhythmia and Long-Term ECG Databases

**Figure 4:**
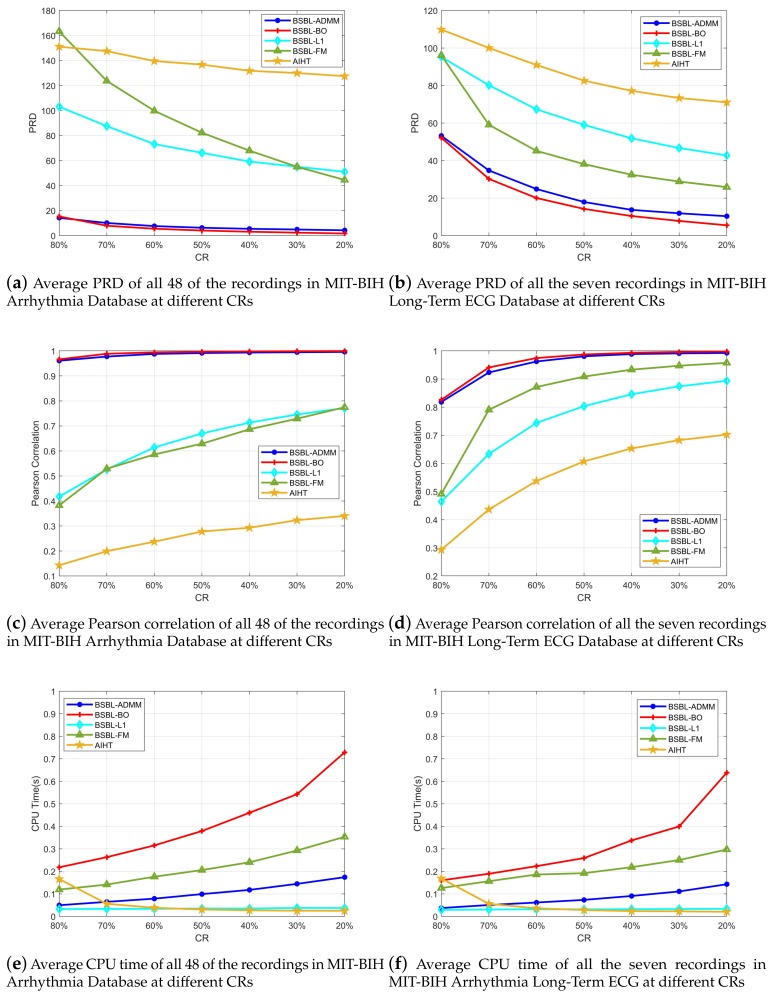
Recovery results of all 48 of the recordings in MIT-BIH Arrhythmia Database and seven recordings in MIT-BIH Long-Term ECG Database at different CRs (compression ratios). (**a**–**d**) show that the PRD (percentage root-mean squared distortion) and Pearson correlation of BSBL-ADMM are very close to BSBL-BO, the other three have much worse recovery accuracy; (**e**,**f**) show that the BSBL-ADMM is much faster compared with BSBL-BO. These results on MIT-BIH Databases demonstrate that the proposed BSBL-ADMM algorithm has both satisfactory speed and accuracy compared with the other algorithms.

In general, real-time wearable ECG telemonitoring has certain requirements of the speed and accuracy of signal recovery, thus the speed and accuracy of the signal recovery algorithm must achieve an acceptable range at the same time. Figure 4 shows the recovery results averaged over all the 48 recordings in the MIT-BIH Arrhythmia Database and seven recordings in the MIT-BIH Long-Term ECG Database. From Figure 4a–d, it can be seen that the proposed BSBL-ADMM algorithm has a very close recovery accuracy relative to BSBL-BO at different CRs, and, when the CR is 80%, the recovery accuracy of the proposed BSBL-ADMM algorithm is even slightly better than BSBL-BO [25]. The other three algorithms BSBL-L1 [25] and BSBL-FM [27] and AIHT [19] have much worse recovery accuracy compared with BSBL-ADMM and BSBL-BO.

In addition, Figure 4e–f shows the average CPU times that the algorithms cost for recovering a single frame of ECG signal, we can see that the speed of the proposed BSBL-ADMM algorithm is much faster than BSBL-BO and BSBL-FM. Although the speed of BSBL-L1 and AIHT is faster than BSBL-ADMM, the recovery accuracy of these two algorithms is very bad. In fact, higher CR usually brings about less CPU time, but the recovery accuracy will be worse, so it is necessary to choose a compromise between recovery accuracy and CPU time according to practical requirements. In our experiments, we choose CR = 60% as the result of a compromise that is acceptable considering both the accuracy and speed, and more detailed results at the CR of 60% will be presented.

**Figure 5:**
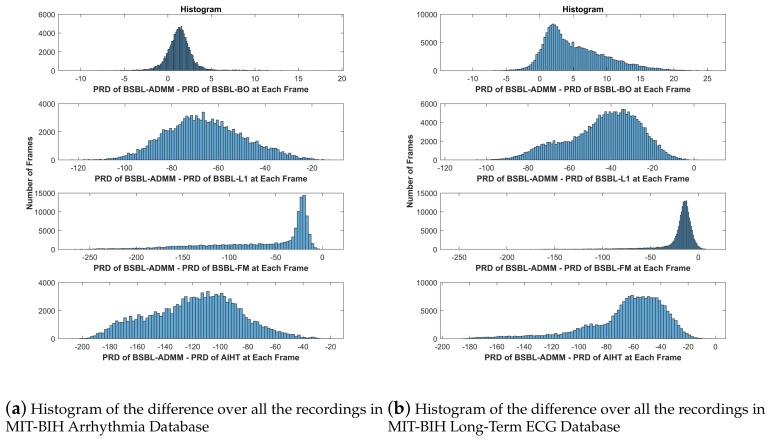
Histogram of the difference between the PRD of BSBL-ADMM and the PRD of the other four algorithms at each frame over all the recordings in MIT-BIH Arrhythmia Database and MIT-BIH Long-Term ECG Database at the CR of 60%. It can be seen that the PRD of BSBL-ADMM is significantly smaller than BSBL-L1, BSBL-FM and AIHT. In addition, the PRD of BSBL-ADMM is larger than BSBL-BO, but the difference is relatively small and the recovery accuracy of the proposed BSBL-ADMM and BSBL-BO is very close.

To better compare the performance of the proposed BSBL-ADMM algorithm with the other four algorithms, Figure 5 plots the histogram of the difference between the PRD of BSBL-ADMM and the PRD of the other four algorithms at each frame over all the recordings in the MIT-BIH Arrhythmia Database and MIT-BIH Long-Term ECG Database at the CR (compression ratios) of 60%, i.e., the histogram of p(i)−q(i), where p(i) indicates the PRD of BSBL-ADMM at the *i*th frame, and q(i) indicates that of BSBL-BO or other compared algorithms at the *i*th frame. The histogram is a statistical comparison of the recovery accuracy of two algorithms. According to the distribution of the difference between PRDs of two algorithms in the histogram, if the part that is less than 0 is in the majority, it means that the former has the better recovery accuracy, and vice versa. In Figure 5, it can be seen that the PRD of BSBL-ADMM is significantly smaller than BSBL-L1, BSBL-FM and AIHT. In addition, the PRD of BSBL-ADMM is larger than BSBL-BO, but the difference is relatively small and the recovery accuracy of the proposed BSBL-ADMM and BSBL-BO is very closed.

**Figure 6:**
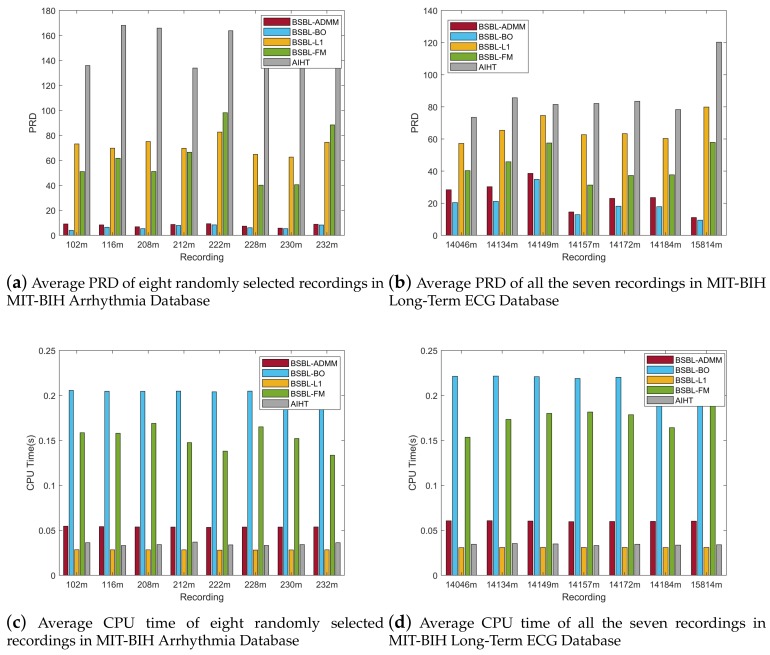
Recovery results of eight randomly selected recordings in MIT-BIH Arrhythmia Database and all seven of the recordings in MIT-BIH Long-Term ECG Database. (**a**,**b**) show that the BSBL-ADMM has relatively stable recovery accuracy for different recordings; (**c**,**d**) show that the the BSBL-ADMM has relatively stable CPU times for different recordings. All these results on MIT-BIH Databases demonstrate the robustness of the proposed BSBL-ADMM algorithm.

Figure 6 demonstrates the robustness of the proposed BSBL-ADMM algorithm. It shows the specific results of eight randomly selected recordings in the MIT-BIH Arrhythmia Database and all seven of the recordings in the MIT-BIH Long-Term ECG Database at the CR of 60%. For each recording, the proposed BSBL-ADMM algorithm has similar recovery accuracy to the average of all recordings. In terms of CPU time, the proposed BSBL-ADMM algorithm has a very stable speed for signal recovery. Therefore, these results show the robustness of the proposed BSBL-ADMM algorithm having a satisfactory performance on different recordings. The robustness ensures that the proposed BSBL-ADMM algorithm is suitable for different ECG datasets in wearable ECG telemonitoring.

Moreover, it can be clearly seen that there is a significant difference in the recovery accuracy of the same algorithm between these two databases. This is mainly because the sampling rates of the two databases are different: the sampling rate of the MIT-BIH Arrhythmia Database is 360 Hz, and the sampling rate of the MIT-BIH Long-Term ECG Database is 128 Hz. Usually a higher sampling rate will bring better recovery accuracy, but the higher sampling rate means more energy consumption of the telemonitoring system. In general, the frequency range of an ECG signal varies between 0.05–100 Hz, thus 250 Hz is a commonly used sampling rate for ECG monitoring in body area networks [35]. We sampled the ECG signal at the sampling rate of 250 Hz in our wearable ECG telemonitoring system for the experiment in the next subsection.

### 4.6. Results on Practical Wearable ECG Datasets

In order to verify applicability of the proposed BSBL-ADMM algorithm in practical application, we built a wearable ECG telemonitoring system described as the above Figure 2 and Figure 3. The practical wearable ECG datasets were collected using our wearable ECG collecting system. The datasets contain eight one-channel recordings collected from eight different people, and every recording was sampled at the sampling frequency 250 Hz and lasted about 30 min. In our experiments, for convenience to calculate the error between the recovered ECG signals and original ECG signals, we transmitted the original ECG signals to the computer and then both the compression and recovery of ECG signals were done on the computer.

**Figure 7:**
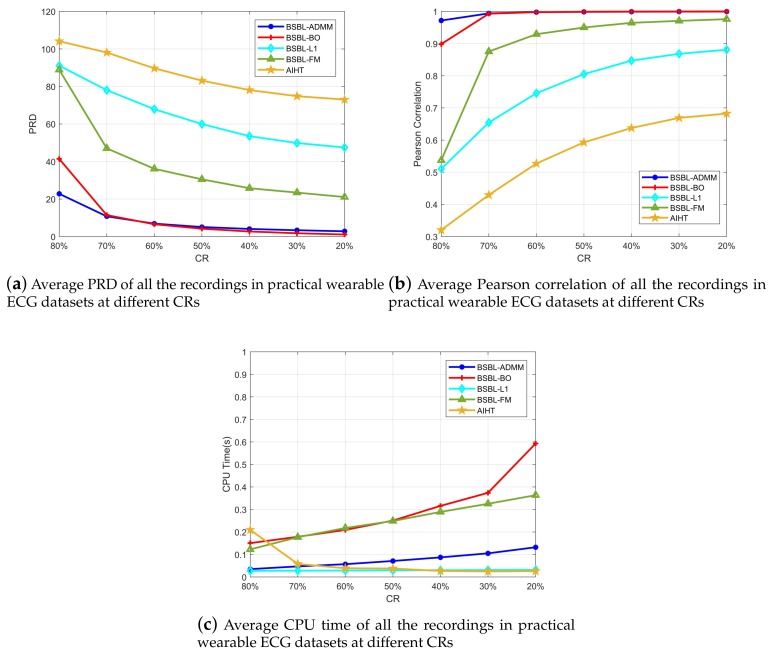
Recovery results of all the recordings in practical wearable ECG datasets at different CRs (compression ratios). (**a**,**b**) show that the PRD (percentage root-mean squared distortion) and Pearson correlation of BSBL-ADMM is very close to BSBL-BO, the other two have much worse recovery accuracy; (**c**) shows that the BSBL-ADMM has much faster speed compared with BSBL-BO. These results on practical wearable ECG datasets demonstrate that the proposed BSBL-ADMM algorithm has both satisfactory speed and accuracy compared with the other algorithms.

Similarly, the experimental results on practical wearable ECG datasets also demonstrate the outstanding performance of the proposed BSBL-ADMM algorithm. The average results of practical wearable ECG datasets are presented in Figure 7. From Figure 7a,b, we can see a very similar result to MIT-BIH Arrhythmia Database that the proposed BSBL-ADMM algorithm has a very close recovery accuracy relative to BSBL-BO, and the other three algorithms have much worse accuracy. Meanwhile, Figure 7c shows the fast speed of the proposed BSBL-ADMM algorithm. Although BSBL-L1 and AIHT have the fastest speeds among these algorithms, both of their recovery accuracies are very bad.

**Figure 8:**
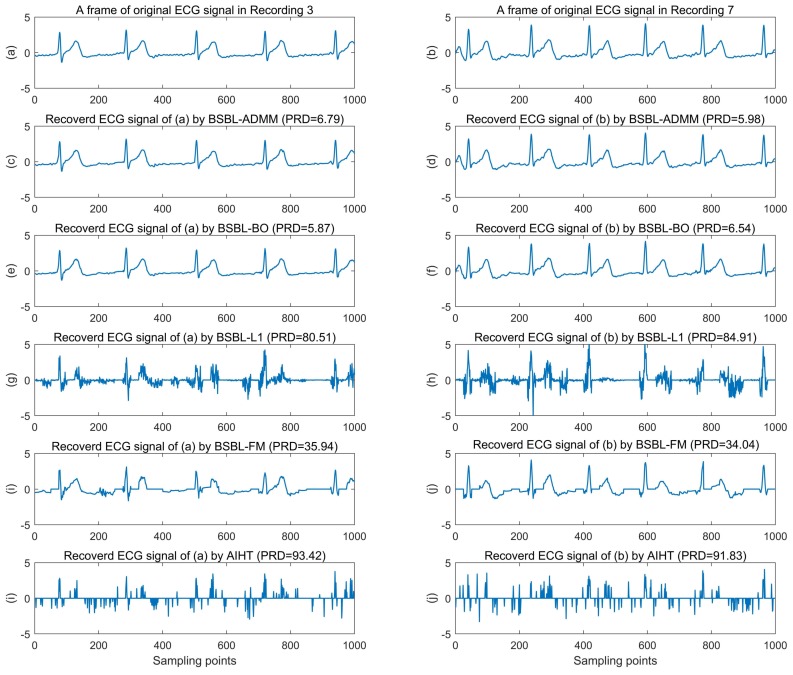
Recovery results of two ECG signals selected from recording 3 and recording 7, respectively. It can be seen intuitively that the recovered ECG signals by BSBL-ADMM and BSBL-BO are very similar and have no obvious distortion compared with the original signal, but the recovered ECG signals by BSBL-L1, BSBL-FM and AIHT are obviously distorted.

To further show the outstanding performance and robustness of the proposed algorithm, we presented the more detailed results of these algorithms on our practical wearable ECG datasets. In order to visually demonstrate the recovery quality of the algorithms, we selected a frame of ECG signal of 1000 sampling points in recording 3 and recording 7, respectively, and presented the original and recovered ECG signals at the CR of 60% in Figure 8. It can be seen that the recovered ECG signals by BSBL-ADMM and BSBL-BO are very similar and have no obvious distortion compared with the original signal, but the recovered ECG signals by BSBL-L1, BSBL-FM and AIHT are obviously distorted.

Moreover, Table 1 shows the details of average PRD and Pearson correlation of every recording in our practical wearable ECG datasets at the CR of 60%. From Table 1, we can see that the recovery accuracy of the proposed BSBL-ADMM algorithm for all eight of the recordings in the practical wearable ECG datasets has little difference, which reflects the robustness. The average PRD and Pearson correlation of the eight recordings are 6.92 and 0.9974, respectively, and the corresponding standard deviations are 0.43 and 0.0003, respectively. Thus, the proposed algorithm is robust to different ECG recordings. Furthermore, Table 2 shows the details of average CPU time of every recording in our practical wearable ECG datasets at the CR of 60%. In addition, it can be seen that the CPU time cost by the proposed algorithm on all eight of the recordings in our practical wearable ECG datasets is relatively stable, the average CPU time of the eight recordings is 0.0629 s, and the standard deviation is 0.0006 s. Therefore, these results demonstrate that the proposed BSBL-ADMM algorithm not only has a fast speed and satisfactory accuracy but also is robust for non-sparse ECG signals in practical wearable ECG telemonitoring systems.

## 5. Discussion

All of these experimental results on the three different datasets demonstrate that the proposed BSBL-ADMM algorithm can recover the non-sparse ECG signals with both satisfactory recovery accuracy and fast speed compared with the other four algorithms. At the same time, the performance of the proposed BSBL-ADMM algorithm is also robust for different datasets. These advantages make the proposed BSBL-ADMM algorithm suitable for real-time ECG telemonitoring.

However, these conclusions are only considered from the perspective of the algorithm itself, and the experiments are carried out on the simulation platform. When the algorithm is actually applied to a practical wearable ECG telemonitoring system, more aspects need to be considered, such as efficient implementation of algorithmic code, the possibility to operate in real time, power consumption and the wireless link. Despite this, the proposed BSBL-ADMM algorithm is still very promising for use in wearable ECG telemonitoring systems, and we will try to actually apply the proposed BSBL-ADMM algorithm to a practical wearable ECG telemonitoring system in our future work.

## 6. Conclusions

In this paper, we proposed a fast and robust non-sparse signal recovery algorithm termed BSBL-ADMM for wearable ECG telemonitoring. This algorithm can recover the non-sparse ECG signals accurately acquired in wearable ECG telemonitoring in real time by using ADMM to accelerate the speed of BSBL framework. Experimental results on the MIT-BIH Databases and our practical wearable ECG datasets showed that the proposed BSBL-ADMM algorithm can recover non-sparse ECG signals fast with a satisfactory accuracy, and it also has robust performance on different ECG datasets. All these advantages indicate that the proposed BSBL-ADMM algorithm will be suitable for wearable ECG telemonitoring.

## Figures and Tables

**Table 1:** The details of recovery accuracy of practical wearable ECG datasets (CR = 60%). These results show that the proposed BSBL-ADMM algorithm is robust for the recovery accuracy for different ECG recordings.

	Algorithms	Rec1	Rec2	Rec3	Rec4	Rec5	Rec6	Rec7	Rec8	Mean (±Std)
**PRD**	BSBL-ADMM	7.34	6.63	6.69	7.63	6.35	6.68	6.82	7.22	6.92 (±0.43)
	BSBL-BO [25]	7.56	6.35	6.18	8.17	5.69	6.42	6.91	6.60	6.74 (±0.80)
	BSBL-L1 [25]	54.10	59.76	78.00	71.60	66.49	60.34	76.37	75.44	67.76 (±8.94)
	BSBL-FM [27]	35.77	34.18	35.10	39.33	35.71	34.88	35.91	38.63	36.19 (±1.82)
	AIHT [19]	101.98	103.74	105.54	106.57	104.40	103.06	103.04	104.55	104.11 (±1.48)
**Pearson Correlation**	BSBL-ADMM	0.9972	0.9977	0.9977	0.9970	0.9980	0.9977	0.9976	0.9974	0.9976 (±0.0003)
	BSBL-BO [25]	0.9970	0.9979	0.9980	0.9966	0.9983	0.9979	0.9976	0.9978	0.9976 (±0.0006)
	BSBL-L1 [25]	0.8436	0.8080	0.6601	0.7236	0.7615	0.8040	0.6804	0.6937	0.7469 (±0.0675)
	BSBL-FM [27]	0.9329	0.9374	0.9321	0.9162	0.9327	0.9359	0.9279	0.9192	0.9293 (±0.0077)
	AIHT [19]	0.4090	0.3961	0.3689	0.3548	0.3669	0.3959	0.3744	0.3657	0.3790 (±0.0190)

**Table 2:** The details of recovery CPU Time of practical wearable ECG datasets (CR = 60%). These results show that the proposed BSBL-ADMM algorithm is robust for the recovery speed for different ECG recordings.

	Rec1	Rec2	Rec3	Rec4	Rec5	Rec6	Rec7	Rec8	Mean (±Std)
BSBL-ADMM	0.0639	0.0635	0.0631	0.0625	0.0625	0.0624	0.0624	0.0628	0.0629 (±0.0006)
BSBL-BO [25]	0.2359	0.2361	0.2353	0.2352	0.2355	0.2350	0.2350	0.2358	0.2355 (±0.0004)
BSBL-L1 [25]	0.0294	0.0293	0.0291	0.0290	0.0291	0.0291	0.0290	0.0291	0.0292 (±0.0001)
BSBL-FM [27]	0.1605	0.2085	0.2868	0.2541	0.2170	0.2038	0.2826	0.2590	0.2340 (±0.0044)
AIHT [19]	0.0458	0.0377	0.0372	0.0444	0.0425	0.0459	0.0407	0.0414	0.0419 (±0.0034)
